# Association sarcoïdose et cirrhose biliaire primitive: à propos d'un nouveau cas

**DOI:** 10.11604/pamj.2014.18.279.1312

**Published:** 2014-08-06

**Authors:** Nourdin Aqodad, Bouchra Loukili, Salim Gallouj, Taoufik Harmouch, Afaf Amarti, FZ Mernissi, Adil Ibrahimi

**Affiliations:** 1 Service d'hépatogastroentérologie, CHU Hassan II de Fès, Maroc, Faculté de médecine et de pharmacie de Fès, Maroc; 2 Service de deratologie, CHU Hassan II de Fès, Maroc, Faculté de médecine et de pharmacie de Fès, Maroc; 3 Service d'anatomopathologie, CHU Hassan II de Fès, Maroc, Faculté de médecine et de pharmacie de Fès, Maroc

**Keywords:** Anticorps anti-mitochondries, choléstase, cirrhose biliaire primitive, sarcoïdose, Anti-mitochondrial antibodies, cholestasis, primary biliary cirrhosis, sarcoidosis

## Abstract

La sarcoïdose est une maladie systémique d’étiologie inconnue. Elle est caractérisée par la présence de lésions granulomateuses, non caséeuses, au niveau des organes atteints. La cirrhose biliaire primitive (CBP) est une hépatopathie cholestatique auto-immune chronique, caractérisée par une destruction des canalicules biliaires et la présence d'anticorps antimitchondries type M2. L'association sarcoïdose et CBP est rare. Nous rapportons un nouveau cas de cette association avec revue de la littérature.

## Introduction

La sarcoïdose est une maladie systémique d’étiologie inconnue. Elle est caractérisée par la présence de lésions granulomateuses, non caséeuses, au niveau des organes atteints. La cirrhose biliaire primitive (CBP) est une hépatopathie cholestatique auto-immune chronique, caractérisée par une destruction des canalicules biliaires et la présence d'anticorps antimitochondrie type M2 [1]. L'association sarcoïdose et CBP est rare, une vingtaine de cas ont été rapporté dans la littérature [2]. L'apport de l'immunologie et de l'anatomopathologiste est déterminant pour différencier l'atteinte hépatique liée à ces deux entités. Dans cet article nous rapporterons un nouveau cas d'association de sarcoïdose et de cirrhose biliaire primitive et nous proposeront une revue de la littérature.

## Patient et observation

Il s'agit d'une patiente âgée de 40 ans dont l'histoire clinique remontait à un mois avant son admission à l'hôpital par l'installation de lésions cutanées érythémato-violacés.

**Figure 1:**
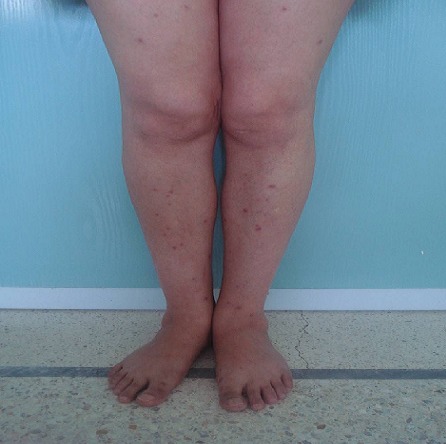
Multiples lésions papuleuses rouge- violacées de taille variables avec la présence d'un infiltrat lupoide à la vitropression, siégeant de façon symétrique à la face d'extension des membres inférieurs

**Figure 2:**
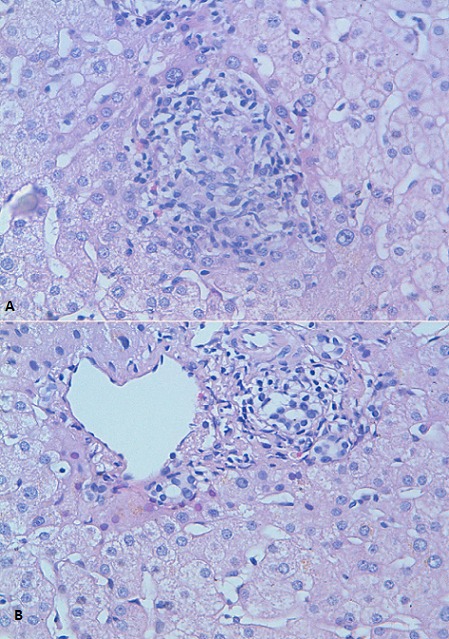
A) granulome épithélioïde associé à quelques polynucléaires éosinophiles. Gx20 HES; B) Espace porte: Prolifération néoductulaire associée à des lésions canalaires à type d'exocytose lymphocytaire. Nécrose parcellaire au niveau de la lame bordante également visible. GX20 HES

Bien limités de taille variable surmontées par de fines squames par endroit siégeant au niveau du nez, du tronc, des deux avant bras et des deux membres inferieurs non prurigineuses (Figure 1), augmentant progressivement de taille associé à une dyspnée stade II sans toux. Cette symptomatologie avait poussé la patiente à consulter chez un dermatologue et une biopsie cutanée était réalisée qui était en faveur d'une dermite granulomateuse. Dans le cadre du bilan de la sarcoïdose on avait réalisé une radiographie thoracique qui avait objectivé un syndrome réticulo-micronodulaire intéressant les deux hémi-champs thoraciques. Le complément TDM thoraco-abdominale avait objectivé une adénomégalie thoracique associée à une atteinte interstitielle de type lymphatique et à une splénomégalie nodulaire (stade II). L'exploration fonctionnelle respiratoire n'a pas objectivé de syndrome restrictif alors que la DLCO était diminuée à 74%. L'examen ophtalmologique avait objectivé un petit nodule palpébral supérieur droit mobile non inflammatoire alors que le reste de l'examen était normal. L'examen cardiovasculaire n'avait pas montré de troubles de conduction. Le dosage de l'enzyme de conversion de l'angiotensine I était augmenté à 147UI/l (N:12-68UI/l). Le bilan phosphocalcique était sans anomalie alors que le bilan hépatique avait objectivé une cholestase anictérique (GGT-1,6N, PA-1,2N). Le cholestérol total était augmenté (2,52 g/l), les transaminases étaient normaux et l’électrophorèse des protides avait montré une augmentation des gammaglobulines. les immunoglobulines M étaient élevées à 1,5N. Dans le cadre du bilan de la cholestase on avait réalisé un bilan immunologique qui avait montré la positivité des anticorps anti mitochondrie à 1/160 type M2 alors que les sérologies virales B et C étaient négatives. L’échographie abdominale avait montré un foie normal. L’étude histologique de la biopsie hépatique avait montré au niveau des espaces portes la présence de granulomes épithélioïdes avec une prolifération néoductulaire (Figure 2 - A) et des lésions canalaires à type d'exocytose par des éléments lymphocytaires associée à quelques polynucléaires éosinophiles (Figure 2 - B). Une discrète nécrose hépatocytaire périportale et lobulaire était associée. Cet aspect était compatible avec une cirrhose biliaire primitive au stade I de Scheuer associée à une sarcoïdose hépatique.

Le diagnostic d'association sarcoïdose systémique associé à une cirrhose biliaire primitif était retenu. La patiente était mise sous Acide Urso-Desoxy-Cholique (15mg/kg), corticothérapie (1mg/kg/j) et antipaludéens de synthèse (nivaquine 200mg/j). Actuellement la patiente est en phase de dégression des corticoïdes avec bonne évolution clinique « disparition des lésions cutanés et de la dyspnée » et biologique « normalisations des PA et de la GGT » avec un recul de 18 mois.

## Discussion

La cirrhose biliaire primitive(CBP) est une maladie auto-immune dont l'incidence annuelle est estimée à 10-20/100000. Elle est diagnostiquée le plus souvent chez la femme à la quatrième ou cinquième décade. Sa prévalence dans les familles de patients ayant une CBP est de 4% [1]. L'atteinte cutanée est révélatrice dans 88% des cas [2]. Le prurit reste le maitre symptôme. Parfois le diagnostic est porté à l'occasion de perturbation des tests hépatiques notamment les transaminases et le bilan de cholestase, comme c'est le cas chez notre patiente. Dans d'autres situations le diagnostic est porté à l'occasion du bilan d'une maladie auto-immune ou au stade de cirrhose dans 10% des cas [1].

La recherche des anticorps anti mitochondries (AMA) est importante dans le diagnostic de la CBP avec une sensibilité de 90% et une spécificité de 95%, les anticorps anti-mitochondrie type M2 ont une spécificité de 100% [3]. D'autres anomalies immunologiques ont été décrites notamment la détection des anticorps antinucléaires et l'augmentation des immunoglobulines(IGM).

La grande majorité des patients ont des tests hépatiques caractérisés par une élévation modérée de l'activité des ALAT et des ASAT, une élévation marquée de l'activité GGT et des phosphatases alcalines.

La ponction biopsie hépatique, non obligatoire pour le diagnostic de la CBP [4], permet essentiellement de stadifier la maladie et de montrer des lésions caractéristiques de cette affection à l'histologie à savoir une cholangite non suppurative affectant les canaux biliaires interlobulaires et septaux. L'infiltrat inflammatoire est composé principalement de lymphocytes et de cellules mononucléées en contact direct avec la membrane basale des cholangiocytes en voie de nécrose. L'inflammation portale peut prendre l'aspect de granulomes épithélioïdes.

La CBP peut être associée à certaines maladies auto-immunes notamment le syndrome de Sjogreen, la sclérodermie, la polyarthrite rhumatoïde, psoriasis, la chondrocalcinose et l'osteodystrophie. L′atteinte pulmonaire dans la cirrhose biliaire primitive est controversée [5]. En effet dans les grandes séries les manifestations pulmonaires ne sont pas communes. Elles sont le plus souvent décrites dans les rapports de cas notamment la pneumonie interstitielle et la fibrose. Weismann Becker avait signalé la pneumonie interstitielle lymphoïde [6], et Davison et Epstein avaient décrit la pneumonie systématisée et le CREST syndrome [7]. Wallaert et al avaient réalisé un lavage broncho-alvéolaire chez 12 patients asymptomatiques avec cirrhose biliaire primitive et radiographies thoraciques normales. Ils ont trouvé que le nombre total de cellules était normal avec augmentation du pourcentage des lymphocytes [8].

La sarcoïdose (maladie de Besnier, Boeck et Schaumann) est une maladie granulomatose diffuse, d’étiologie inconnue, qui peut toucher de nombreux organes: le poumon préférentiellement (localisation médiastino-thoracique) mais aussi la peau, la synoviale, l'os, le c'ur, le rein ou le système nerveux central et le foie. L'atteinte respiratoire est présente chez 95-98%des malades [9, 10].

L'atteinte médiastino-thoracique est isolée dans 40% des cas ou s'associe à des manifestations extra-thoraciques dans 40% des cas. Les formes extra-thoraciques isolées sont plus rares (environ 20%)[11]. Elle survient préférentiellement entre 20 et 40 ans et est plus sévère chez le sujet Noir. La glande hépatique est atteinte au cours de la sarcoïdose systémique dans deux tiers des cas. L'hypertension portale (HTP) et la cirrhose ne sont observées que dans 1% des cas des séries de sarcoïdose. Cependant, la prévalence de l'HTP au cours de la sarcoïdose serait de 30 à 45% chez les patients symptomatiques [12]. Le diagnostic de la sarcoïdose est porté par l'intrication de 03 arguments:

Présentation clinique, radiologique et biologique évocatrice Mise en évidence d'un granulome epitheliogiganto cellulaire au moins sur un prélèvement histologique.

Exclusion d'une autre maladie granulomateuse notamment dans notre contexte la tuberculose.

L'association de la sarcoïdose systémique à la CBP reste rare. La revue de littérature dénombre vingt cas bien documentés (Tableau 1). Ce sont deux granulomatoses hépatiques dont le diagnostic différentiel est parfois très difficile à établir en pratique. Cependant, certains paramètres cliniques, biologiques, immunologiques et histologique permettent d'orienter le clinicien (Tableau 2). Le granulome hépatique est rencontré dans la sarcoïdose dans 15 à 65% des patients, celui-ci est caractérisé par une structure concentrique, bien délimité, nombreux et localisé au niveau de la zone portal et periportale. Alors que les granulomes dans la CBP sont caractérisés par leur petit nombre, ils sont mal définis et associés à un infiltrat lymphocytaire adjacent aux lésions biliaires. Concernant l'infiltrat lymphocytaire des granulomes dans les deux maladies montrent une accumulation de cellules T CD4 (auxiliaires) dans le centre du granulome. Alors que les cellules T CD8 (cytotoxiques) sont des cellules vues à la périphérie de granulomes sarcoïdosiques et près des voies biliaires dans la CBP [2, 10].

**Tableau 1:** Caractéristiques épidemiologiques des cas rapportés dans la littérature de l'association cirrhose biliaire primitive et sarcoidose (20 cas bien documentés)

Sexe	19F/1H
Age moyen (extrêmes)	60 ans +/- 18 (30-73 ans)
Sarcoidose cutanée + CBP	9 cas
Sarcoidose systémique + CBP	11 cas
**Diagnostic de la CBP**	
AMA +	16 cas
AMA type M2 +	4 cas
Ponction biopsie hépatique :	19/20
Signes de CBP+ granulomes	13 cas
Signes de CBP sans granulomes	5 cas
Cirrhose	1 cas

**Tableau 2:** Tableau comparatif des caractéristiques de la cirrhose biliaire primitive et de la sarcoidose

	caractéristiques	cirrhose biliaire primitive	sarcoïdose
**épidémiologie**	Sex-ratio	Prédominance féminine	1
**Symptômes**	Prurit	Oui	Non
	Ictère	Oui	Rare
	xanthomes	Oui	non
**Biologie**	Cholestase	oui	rare
	AMA	Oui >90%	Non
	IgM	Élevées	Normale
	ECA	<20%	40-50%
**imagerie**	ADP hile pul	Non	Oui
	Anomalies EFR	non	oui
**histologie**	Granulomes	Moins nombreux	Nombreux
	Nombre	Mal défini associé à	Bien défini
	Aspect	infiltrat lymphocytaire	Portale et périportale
	Localisation	Adjacents aux	
	ductopénie	Lésions biliaires	non
		oui	

Dans notre cas le diagnostic de sarcoïdose était posé sur la présentation clinico-biologique, radiologique et la biopsie cutanée. Le diagnostic de la CBP était retenu sur la présence d'une choléstase, la positivité des anticorps anti-mitochondrie type M2 et la présence de signes spécifique de la maladie sur la PBH. Kishor et al avait récemment rapporté 17 patients atteints de sarcoïdose et CBP et a suggéré qu′une voie commune contribue à la formation de granulomes dans les deux affections [2], [13]. Une explication pour l′association apparente entre ces conditions est que les deux partagent le même défaut dans l′immunité à médiation cellulaire [2, 3, 14].

## Conclusion

La CBP et la sarcoïdose ont en commun certaines caractéristiques cliniques, biologiques et histo-morphologiques. Leur étiologie reste inconnue. Cependant leur association suggère une origine commune du granulome.
